# The use of diagnostic coding in chiropractic practice

**DOI:** 10.1186/s12998-015-0051-1

**Published:** 2015-02-18

**Authors:** Cecilie D Testern, Lise Hestbæk, Simon D French

**Affiliations:** Nordic Institute of Chiropractic and Clinical Biomechanics and Institute of Sports Science and Biomechanics, University of Southern Denmark, Odense, Denmark; General Practice and Primary Health Care Academic Centre, University of Melbourne, Melbourne, VIC Australia; School of Rehabilitation Therapy, Faculty of Health Sciences, Queen’s University, Kingston, Ontario Canada

**Keywords:** Diagnostic coding, Chiropractors

## Abstract

**Background:**

Diagnostic coding has several potential benefits, including improving the feasibility of data collection for research and clinical audits and providing a common language to improve interdisciplinary collaboration. The primary aim of this study was to determine the views and perspectives of chiropractors about diagnostic coding and explore the use of it in a chiropractic setting. A secondary aim was to compare the diagnostic coding undertaken by chiropractors and an independent coder.

**Method:**

A codin exercise based on the International Classification of Primary Care version 2, PLUS extension (ICPC-2 PLUS) provided the 14 chiropractors with some experience in diagnostic coding, followed by an interview on the topic. The interviews were analysed thematically. The participating chiropractors and an independent coder applied ICPC-2 PLUS terms to the diagnoses of 10 patients. Then the level of agreement between the chiropractors and the coder was determined and Cohen’s Kappa was used to determine the agreement beyond that expected by chance.

**Results:**

From the interviews the three emerging themes were: *1) Advantages and disadvantages of using a clinical coding system in chiropractic practice, 2) ICPC-2 PLUS terminology issues for chiropractic practice* and *3) Implementation of a coding system into chiropractic practice.* The participating chiropractors did not uniformly support or condemn the idea of using diagnostic coding. However there was a strong agreement that the terminology in ICPC-2 PLUS would not be applicable or desirable for all practice types. In the coding exercise the chiropractors in total coded 202 diagnoses for 135 patients. The overall percentage agreement between the chiropractors and the coder was 52% (17% expected by chance) with a Kappa score of 0.4 (95% CI 0.3-0.7). Agreement was lower for more detailed coding (percentage agreement 35%; Kappa score of 0.3 (95% CI 0.2-0.5)).

**Conclusion:**

It appears that implementation of diagnostic coding would be possible in the majority of the chiropractic practices that participated in this study. However for those chiropractors who do not focus on symptoms in their approach to clinical care, it could be challenging to use the ICPC-2 PLUS coding system, since ICPC-2 PLUS is a symptom-based classification.

**Electronic supplementary material:**

The online version of this article (doi:10.1186/s12998-015-0051-1) contains supplementary material, which is available to authorized users.

## Background

Diagnostic coding in chiropractic practice has several potential benefits. These benefits include improving the feasibility of data collection for research and clinical audits, improving the clinical applicability of research, and providing a common clinical language to help improve interdisciplinary collaboration. Diagnostic coding allows for systematic classification of clinical information in clinical practice and assists with the conduct of clinical audits and data collection for research purposes. Use of the same classification system by different clinical groups creates a common language between different healthcare professions and sectors and could enhance interdisciplinary collaboration. This could lead to better communication, improved continuity of care as well as simplifying the process of referring patients [1].

The International Classification of Primary Care (ICPC) is an example of a diagnostic coding system that could be relevant for chiropractic practice [2]. The ICPC consists of 17 chapters based on body systems following the principle that localisation has precedence over aetiology. A second version of the diagnostic code system, International Classification of Primary Care, Version 2 (ICPC-2), was published in 1998. Originally ICPC was designed for paper based data collection and analysis, but it has spread to electronic clinical and research systems. ICPC has gradually received increasing recognition and use, especially in Europe and Australia [2].

Diagnostic coding is not commonly undertaken in chiropractic practice, and the choice of an appropriate coding system is important as the profession moves towards this. A study in Danish general medical practice showed that the ICPC has a good reliability and validity at the chapter level for musculoskeletal conditions [3]. Thus, it would appear that ICPC is a good choice for diagnostic coding in chiropractic practice since most conditions treated are musculoskeletal [4]. Additionally, the ICPC-2 is already integrated in several healthcare systems [3], as well as into the International Classification of Disease (ICD-10), a coding system used in hospital settings [2].

ICPC-2 classifies clinical information using three character codes called a *rubric* [5]. The first character, a letter, represents a chapter or a body system (such as Musculoskeletal, Cardiovascular or Neurological), and the two additional characters, a number, represent a concept within this body system (a symptom/complaint, problem/diagnosis or process of care). To allow for greater detail and specificity, the ICPC-2 PLUS terminology was developed. ICPC-2 PLUS is a clinical terminology (*terms*) classified to the ICPC-2. ICPC-2 PLUS has been developed for the Bettering the Evaluation And Care of Health (BEACH) research project, which is an ongoing Australian national study of general medical practice activity [6]. ICPC-2 PLUS is also used in Australia in age-sex disease registers, morbidity registers and electronic health records in primary care.

New ICPC-2 PLUS codes are created by aligning a *term* with the description of the specific problem/diagnosis, or type of care, with the most appropriate ICPC-2 *rubric*. For each of the ICPC-2 PLUS *terms* a three digit code is assigned. This provides ICPC-2 PLUS with a six-character identifier as oppose to the three digits identifier for ICPC-2. As such, the final three digits of the six character ICPC-2 PLUS code identify the specific *term* within the *rubric*. For example, the three character ICPC-2 code L01 represents ‘Neck Symptom or Complaint’. In ICPC-2 PLUS there are 11 neck-related *terms* in the L01 rubric to describe patient problems. For example ‘Pain;neck’ is represented in ICPC-2 PLUS as L01 004 and the ‘Cervicalgia’ as L01 006 [2].

Knowledge of who receives chiropractic care and what care they receive is important to understand contemporary chiropractic practice [3]. The Chiropractic Observation and Analysis STudy (COAST) [4] was a cross sectional observational study that described the clinical practice of chiropractors in Victoria, Australia. COAST explored why people seek chiropractic care, what diagnoses chiropractors make and what treatment chiropractors provide. The research method used in COAST was based on the BEACH study methods [6]. In coding the chiropractic clinical information, some new *terms* were required to describe chiropractic clinical practice. In developing the chiropractic specific ICPC-2 PLUS (Chiro), researchers followed the BEACH coding rules [7].

The long-term aim of this area of work is to implement diagnostic coding into chiropractic clinical practice. We are not aware of any previous research exploring the use of diagnostic coding in the chiropractic profession. This project explored chiropractors’ attitudes towards diagnostic coding to provide important information about how coding will be received in this setting. Specifically, the primary purpose was to investigate the views among practising chiropractors in relation to feasibility, applicability and future perspectives of diagnostic coding in chiropractic clinical practice. A secondary aim was to compare the diagnostic coding undertaken by chiropractors to that undertaken by an independent coder.

## Method

### Summary

This study consisted of quantitative and qualitative components. Each of the chiropractors took part in a coding exercise and this exercise then formed the basis for the subsequent interview. The coding exercise primarily provided the chiropractors with some experience in diagnostic coding for them to describe in the interview. The coding exercise also provided the opportunity to compare the diagnostic coding undertaken by chiropractors to diagnostic coding undertaken by an independent coder. We aimed to determine how accurate the coding process was by determining if an independent coder used the same codes, (in this case *terms*) that chiropractors used, considering the chiropractors had more information available to them. The interviews were analysed qualitatively and the coding exercise was analysed quantitatively.

### Participants and recruitment

Chiropractors approached for this study had all participated in the COAST project in 2011 [4] and were currently in clinical practice in Victoria. Chiropractors had already received training in completing the COAST encounter forms and they were familiar with the background for the study. Fifty-two chiropractors participated in COAST and they were all approached for this study. The chiropractors received an invitation pack in the post, including an invitation letter for the study, a Plain Language Statement describing what participation in the study consisted of, and a consent form. Chiropractors opted into the study by returning a signed consent form via post or fax/email.

After two weeks the chiropractors who had not responded to the invitation letter were sent a follow up letter. After two additional weeks, non-responders were contacted by telephone until 15 chiropractors agreed to participate.

Patients were invited to participate in the study when they attended the practices of participating chiropractors during the recording period. Patients were approached if they were 18 years or older and had spinal pain (neck or back pain). Patients received a Plain Language Statement with information about the study and the chiropractors were trained to obtain verbal consent from patients wishing to participate.

### Sample size

The number of chiropractors chosen (15) was by convenience. This number of interviews was feasible to complete considering resources and time available. Further, we are not aware of any similar studies conducted in this setting so this preliminary data would provide important information that can be used for future, larger studies.

For the quantitative analysis, we assumed the agreement on the choice of codes between the coder and the chiropractors would be around 50% with an intra-cluster correlation of 0.1. Therefore a sufficient sample size to provide a 95% confidence interval with a margin of error of 11% was 150 encounters or 10 patients per chiropractor.

### Data collection and analysis

First, the 15 chiropractor participants were asked to complete encounter forms for 10 consecutive patients with neck or back pain. The encounter form used for the data collection was a slightly modified version of the encounter form used in COAST and included patient demographics, up to three reasons for encounter, relevant health information, presenting pain, diagnosis and care given (see Additional file 1 for a copy of the form).

After the chiropractor had completed their patient data collection, face-to-face semi-structured interviews were conducted between the chiropractor and the first author (CT). During the interviews the first author presented the chiropractors with a short introduction to ICPC-2 PLUS. Chiropractors were then asked to choose the ICPC-2 PLUS *terms* most relevant for the diagnoses of the patients. In order to make the coding decision, the chiropractors were asked to use their memory of the patient encounter, the patient’s clinical file and the encounter recording form they had completed at the time of the consultation. The chiropractor could document up to three diagnoses for each patient encounter and therefore also choose up to three ICPC-2 PLUS *terms* per patient encounter.

To make the coding exercise feasible and relevant to back and neck pain problems/diagnoses, the chiropractor was provided with a shortened list of ICPC-2 PLUS *terms* to choose from. The list only included *terms* relevant to neck and back pain from the ICPC-2 PLUS musculoskeletal chapter (254 *terms*).

The face-to-face interview consisted of open-ended questions about the chiropractors’ perspective and views on the use of diagnostic coding in chiropractic practice. The questions enquired about the chiropractors’ views on diagnostic coding, the potential role of a diagnostic coding system in chiropractic practice, the chiropractors’ views on using the ICPC-2 PLUS *terms* in chiropractic practice, and finally the facilitators and barriers the chiropractors faced when trying to use ICPC-2 PLUS. All interviews were audio recorded and transcribed verbatim.

The patient encounter forms completed by the chiropractors, together with the chiropractors’ choice of ICPC-2-PLUS *terms*, were collected at the end of the interviews. An independent hospital trained coder examined the encounter forms and assigned relevant ICPC-2 PLUS *terms* to the diagnosis recorded by the chiropractor on the forms. The coder used the “Demonstrator” function on the ICPC-2 PLUS website to select the appropriate *terms* for the diagnosis provided by the chiropractors [8]. The Demonstrator is an online search tool that allows access to all of the ICPC-2 PLUS *terms*. This coder searched an extensive keyword list with keywords linking the ICPC-2 PLUS six-character codes. When selecting a keyword, the coder was presented with all the available associated *terms*. The coder then selected the *term* that most closely reflected the chiropractors’ exact wording of the diagnosis written on the encounter forms. The only information available about the patient for the coder was the information on the encounter forms, as is the case for the BEACH and COAST studies. Encounters were excluded from agreement analysis where the ICPC-2 PLUS *term* chosen by the coder was not included on the list provided to the chiropractors.

For the qualitative analysis, all interviews were examined by the first author (CT) by reading through and listening to each of the transcripts/audio files and labeling paragraphs with descriptive and interpretive codes as suggested by King [9]. After the first read through commonly repeating patterns were identified for each transcript. The same process was performed by the third author (SF) for the interview transcripts of three chiropractors, each with a different type of clinical practice. Descriptive and interpretive codes were then compared between the first and third author for the three interview transcripts, and divergent views between the two authors were resolved through discussion. The first author then re-examined the remaining 11 transcripts in light of this discussion.

For each of the interviews the repeating patterns were merged to define broader themes [10]. The interview were then finally analysed by looking for common themes across all of the 14 interviews, as well as areas of differences in the views and perspectives of the participants [11].

For the quantitative analysis of the coding exercise, the agreement between the independent coder and the chiropractors for their choices of ICPC-2 PLUS *terms* was determined at two levels: ICPC-2 *Rubric level*, three-character code; and also ICPC-2 PLUS *term level*, six-character code. The analysis was undertaken at two levels to determine whether more specific coding at the *term level* resulted in lower agreement. The overall percentage agreement was calculated, as well as the percentage agreement for each practitioner. Cohen’s Kappa was used to determine the agreement beyond that expected by chance, using the statistical software Stata. Kappa was derived by comparing whether each chiropractor and the coder agreed (or not) on the choice of codes, and a mean Kappa value with 95% confidence intervals was determined. The guidelines of Landis and Koch [12] were used for interpreting the Kappa values. Finally, the time taken for the chiropractors to make a choice about a diagnostic code was determined from the audio recordings.

### Ethics approval

The project received full approval from the Human Ethics Subcommittee at the University of Melbourne on 20th of June 2012 (ID1237727) as a Minimal Risk Project following the ethical guidelines described by the Australian National Health and Medical Research Council, NHMRC [13]. All participants (chiropractors and patients) gave informed consent to participate.

## Results

Fourteen chiropractors completed the study and their demographic characteristics are described in Table 1. One chiropractor did not have the encounter forms available at the time of the coding exercise and was therefore not included in the study. Most of the chiropractors were males practising in metropolitan Melbourne. All participated in the COAST project, but typically did not have any other experience participating in research.

**Table 1:** **Characteristics of participating chiropractors**

**Chiropractor characteristics (N = 14)**		
**Chiropractor characteristics**	**Average**	***Range***
Age in years	46	30-57
Years in practice	19	5-32
	**No.**	***%***
Gender		
Female	1	7
Male	13	93
Location		
Metropolitan*	10	72
Rural	4	28
Graduated in Australia	13	92
Holds Postgraduate qualification	5	35
Involved in Teaching	3	20
Membership:		
CAA	6	42
COCA	5	35
Other **	6	42
Computer use in clinic:		
Do not have computer in practice	2	14
Clinical records paperless	1	7
Clinical records partially paperless	5	35
Clinical records paper only	7	50

*Determined from Australian Standard Geographical Classification - Remoteness Area (ASGC-RA).

**Other includes: The Australasian College of Chiropractors, Gonstead Chiropractic group, Australian Spinal Research Foundation, CAA sports.

CAA = Chiropractors’ Association of Australia; COCA = Chiropractic and Osteopathic College of Australasia.

The characteristics of the 135 participating patients are described in Table 2. The majority of the patients were between 46 and 70 years of age. There were more female than male patients but the difference in the distribution was only 10% (4 missing). Most patients (58%) presented with one reason for encounter and the most common reason was back pain.

**Table 2:** **Patient demographics and encounter reasons**

**Patient characteristics (N = 135)**	**No.**	**%**
**Age** [missing]	[7]	5
18-30	24	18
31-45	38	28
46-70	61	45
70+	5	4
**Gender** [missing]	[5]	4
Male	58	43
Female	72	53
**Number of reasons for encounter per patient:** [missing]	[3]	2
1	77	57
2	40	30
3	15	11
**Reasons for encounter:** [missing]	[7]	5
Neck pain	46	22
Back pain	76	38
Check up/maintenance	27	13
Other*	53	26
**Total number of reasons for encounter**	202	
**Total number of diagnoses provided by the chiropractors**	167	

*Examples of other: Headache, shoulder pain, arm pain, leg pain.

Note: The number of patients, the number of reasons for encounter and the number of diagnoses varied. Patients could report up to three reasons for encounter, which meant that some patients could have multiple diagnoses. In other cases the chiropractor combined some of a patient’s reasons for encounter into one diagnosis thus for these patients there could be more reasons for encounter than diagnoses.

### The interview themes

Three overall themes emerged from the interview data: *1) Advantages and disadvantages of using a clinical coding system in chiropractic practice, 2) ICPC-2 PLUS terminology issues for chiropractic practice, and 3) Implementation of a coding system into chiropractic practice.* The themes are described in the following section along with illustrative quotes from the participating chiropractors. For additional quotes see Additional file 2.

### Theme 1) Advantages and disadvantages of using a clinical coding system in chiropractic practice

Most of the chiropractors were positive towards to the idea of using diagnostic coding in chiropractic practice and they suggested many possible advantages. Some talked about how this could streamline their own clinical practice. Many suggested the possibility of creating a database of coded clinical practice information. They suggested that this database could be used to produce research more efficiently and to ensure the clinical relevance of the research produced.

However, some of the chiropractors did not see the relevance or purpose of ICPC-2 PLUS in chiropractic practice. An issue mentioned by many was that the terminology would not fit into what they called the “wellness-based approach” of some chiropractors. Some chiropractors suggested that the use of ICPC-2 PLUS in chiropractic practice could “medicalise” the profession. On the other hand many chiropractors discussed how diagnostic coding could create a common language that could facilitate better communication between the different musculoskeletal health professions. Some chiropractors also mentioned how this could lead to integration into the mainstream healthcare system. Some illustrative quotes demonstrating these issues follows:

*“The advantages are quite evident, what it can create is a very good database for epidemiological studies and for further research and getting a better appreciation of the type of presentations and you got a lot of data that then could be stored, collected and accessed and drawn upon for future research”* [ID1, 48 years old, 16 years in practice, Metro].

*“The minute I start doing this* [coding exercise] *with trying to match their reported reason for encounter that then assumes that what you are doing on that day is a treatment for that reason for encounter right? And I can understand why you would have this if you are in medicine because that is what they do right? But that’s not what we do. So someone might have a presenting complaint: “okay let’s check you, let’s see if you are subluxated, if you are then I’ll adjust you, whether or not you’ve got that presenting complaint, it’s irrelevant okay.. So I don’t know how you could fit this* [ICPC-2 PLUS] *into the way I have just explained it, if there was a way that would be great.”*[ID8, 43 years old, 19 years in practice, Metro].

*“If it’s going to create a language that helps through - not just the chiropractic profession communicating well with each other, but chiropractic profession communicating with the rest of the medical industry - medical - well, rather, the health world. Then that’s good for everybody. So, that’s a great role and if we’re talking about chiropractic - getting a larger level of research towards it and integrating itself into a mainstream system more. I think that would be actually essential. So, yeah there’s certainly a role for it.”*[ID13, 30 years old, 5 years in practice, Metro].

### Theme 2) ICPC-2 PLUS terminology issues for chiropractic practice

The terminology in ICPC-2 PLUS was a topic mentioned frequently by the chiropractors. Many of the chiropractors mentioned that there were too many *terms* to choose from, but also that the *terms* were not specific enough to reflect chiropractic clinical practice. They believed that there were not enough relevant *terms* for the stage of the problem/diagnosis, for example the limited possibilities to describe if a problem/diagnoses was acute or chronic or somewhere between the two. Some illustrative quotes demonstrating these issues follows:

*“Obviously they are not specific enough, it doesn’t tell you the state of whether it’s acute, chronic or what have you. Again it might tell you the region you are looking at but not the anything specific of what you are looking at”* [ID2, 50 years old, 28 years in practice, Metro]

*“It wasn’t easy cause 27000 things for the same thing (laughs) almost, yes too many choices and not specific enough or they are trying to be too specific which then makes it a bit harder to find exactly what is going on.”* [ID6, 46 years old, 11 years in practice, Rural]

### Theme 3) Implementation of a coding system into chiropractic practice

Many chiropractors thought that implementation of ICPC-2 PLUS into chiropractic clinical practice would be feasible after some practice. Some of the issues mentioned were getting ICPC-2 PLUS into the existing electronic clinical record systems and that not all chiropractors use electronic clinical practice records. Some chiropractors mentioned that implementing a system like ICPC-2 PLUS would have to start at the undergraduate level. One chiropractor stated that the idea was good but the motivation for clinicians was not great enough to make implementation of ICPC-2 PLUS possible. Some illustrative quotes demonstrating these issues follows:

*It got easier. I guess part of it is just being familiar with what your options are.* “ [ID12, 38 years old, 16 years in practice, Rural]

“*When you familiarize yourself with it, it would be great.*”[ID1, 48 years old, 16 years in practice, Metro]

“*I really think it’s gotta start at the undergraduate level. To start teaching people this is the way of the future. And through training sessions and seminars disseminating it to the field practitioners*.”[ID7, 49 years old, 25 years in practice, Metro]

### Coding exercise and agreement analysis

The 135 included patients provided 202 reasons for encounter. The chiropractors spent between 30 seconds to 20 minutes choosing a code for a particular diagnosis/problem.

**Figure 1:**
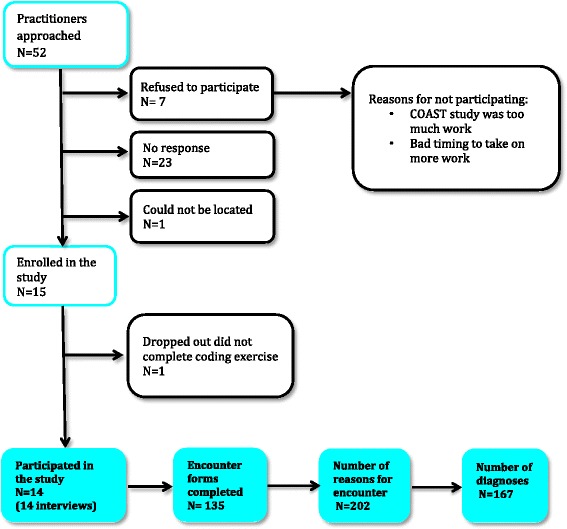
**Flow of participants and data in the study.**

Each of the 14 participating chiropractors recorded 10 patient encounters. For each of these patient encounters, the chiropractor could list up to 3 diagnoses. The total number of diagnostic codes provided by the chiropractors, and used in the agreement analysis, was 167 (Table 3). See Figure 1 for flow of participant and data in the study.

**Table 3:** **Level of agreement**

**Chiropractor ID**	**Number of diagnoses coded**	Agreement on * ***rubric level*** **(%)**	Agreement on ** ***term level*** **(%)**
1	9	89	67
2	20	15	10
3	12	92	50
4	15	60	0
5	14	29	29
6	9	67	56
7	14	29	7
8	10	100	100
9	16	44	31
10	11	82	73
11	10	100	10
12	11	27	18
13	12	0	0
14	4	75	0
**Overall agreement %**		52	35
**Agreement expected by chance**		17	6
**Overall agreement (Cohen’s Kappa)**	**Total number of codes:** 167	0.4 (95% CI 0.3-0.7)***	0.3 (95% CI 0.2-0.5)***

*Rubric level: ICPC-2 classifies symptoms/complaints, problems/diagnoses and process of care using three character codes called a rubric. The first character, a letter, represents a chapter or a body system and the two additional characters, a number, represent a concept within this body system.

**Term level: To allow for greater specificity, the ICPC-2 PLUS terminology was developed. Each PLUS *term* is classified to ICPC-2. ICPC-2 PLUS uses a six-character identifier by adding another three digits (a code) to the ICPC-2 rubric, to which the *term* has been classified.

***CI = 95% confidence interval.

As presented in Table 3 the overall percentage agreement between the coder and the chiropractors on the ICPC-2 *Rubric level* (three-character code) was just over 50% agreement. On ICPC-2 PLUS *term level* (six-character code) the agreement was just over 30%. The level of agreement beyond chance was moderate with a Kappa score 0.4 (95% CI 0.3-0.7) on the ICPC-2 *Rubric level*, and fair with a Kappa score 0.3 (95% CI 0.2-0.5) on the ICPC-2 PLUS *term level*.

## Discussion

This paper provides a first insight into views and perspectives of chiropractors about diagnostic coding in chiropractic practice. ICPC-2 PLUS was chosen as the example of a diagnostic coding system in this study because it has been proven to have a good reliability and validity for musculoskeletal diagnosis, which is the most common conditions treated in chiropractic practice. Also, it is already used in hospital settings/ secondary sector, the COAST data was coded with this particular classification and alongside the process a set of *terms* for chiropractic practice is under development ICPC-2 PLUS (Chiro) [7].

Most chiropractors were positive toward using a diagnostic coding system in their daily practice but some of those chiropractors did not see the relevance of the ICPC-2 PLUS for their practice. The chiropractors who described themselves as *wellness chiropractors* typically expressed the view that the terms available were not adequate to describe their clinical practice. The main issue here was the fact that they did not have a particular focus on specific symptoms. They said that it was obvious that ICPC-2 PLUS terms were made for medical practice, and that it would be better to make a new system for chiropractic practice with more relevant terms. Coding with the current terms in ICPC-2 PLUS may therefore not be relevant for all practice types. The terminology issue was also addressed alongside the conduct of COAST and resulted in a chiropractic-specific coding system being developed, ICPC-2 PLUS (Chiro) [7]. Many however agreed that with some training, ICPC-2 PLUS might be a useful system to have in chiropractic practice, especially to facilitate producing more clinically relevant research.

For the coding exercise if agreement between the chiropractor and coder was high, this would provide some support that ICPC-2 PLUS was potentially a good choice for diagnostic coding in chiropractic practice. If the agreement was low it would tell us that something needed improvement. Either ICPC-2 PLUS was not a good fit for chiropractic practice, or the encounter forms the chiropractors completed did not provide enough information for the coder to agree with the chiropractors’ choice of code.

The agreement between the coder and the chiropractors was higher than agreement expected by chance at both the ICPC-2 *Rubric level* and the ICPC-2 PLUS *term level*. However, the agreement was only fair to moderate and this could be due to a number of reasons. First, none of the chiropractors were familiar with the *terms* available for coding when they completed the encounter forms. The amount of time spent to get familiar with the *terms* available before the coding exercise differed considerably. The practitioners spent between 30 seconds to 20 minutes choosing a *term* for a particular diagnosis/problem. Some of the participating chiropractors mentioned that they would have preferred some prior knowledge about the available *terms* before filling out the encounter forms. To avoid adding to the burden for the participating chiropractors, we decided not to include the list of *terms* in the study materials they received prior to the interviews. We do not know whether this would have improved the level of agreement or had an influence on their overall view on the idea of diagnostic coding in chiropractic practice.

For the agreement on ICPC-2 *rubric* level two chiropractors had 100% agreement with the coder, eight chiropractors had over 50% agreement with the coder. But the two chiropractors who achieved 100% agreement with the coder had only chosen one *term* for all the diagnoses. Despite a variation in their patients’ reasons for encounter, the diagnoses, written by the chiropractor on the encounter form, were all the same. All other chiropractors had used at least three different *terms* when coding. In order to conduct the agreement analysis the participating chiropractors had to choose a *term* for every one of their diagnoses. They were asked to choose the *term* that came closest to describing the diagnosis, rather than being given an option of not choosing a *term*. This might have resulted in these two chiropractors choosing the same *term* for all the diagnoses.

Even the chiropractor who did not agree with the coder on any of the *terms* chosen was positive to using ICPC-2 PLUS in practice. Another chiropractor had an 82% agreement rate with the coder but was negative to the idea of using ICPC-2 PLUS in practice and emphasized a lack of relevance of the coding system to their clinical work and therefore lacked of motivation to implement the system. This chiropractor did not have a wellness practice.

### Limitations and strengths

The sample size for both chiropractors and patient participants were smaller than planned. We did not reach our intended number of practitioners (14 instead of 15) and therefore also less patient participants. This meant one interview less than expected and less data for the agreement analysis. However, this study being the first of its kind and therefore exploratory we do not know what effects this would have had on the results. Qualitative research is not meant to be representative, it is meant to provide in-depth information and this study offers some insight to what possibilities and challenges diagnostic coding could bring to chiropractic practice.

The qualitative interviews, as well as the analysis, were completed with an awareness of the first author’s (CT) personal background as a chiropractic student with minimal clinical experience, and a preconception that diagnostic coding could potentially improve patient care. Being aware of this a neutral approach was emphasized when interviewing and when conducting the analysis.

### Suggestions for further studies

This study was the first to examine the role of diagnostic coding using an established primary care coding system in chiropractic practice. To determine the applicability of ICPC-2 PLUS or another diagnostic coding system in chiropractic practice, more research is needed. A study with a bigger sample would provide further information on this topic.

In Denmark an introduction of the Danish version of ICPC-2, ICPC-2-DK to chiropractic practice is currently underway. ICPC-2-DK has been part of the electronic medical records in Danish general medical practice since 2009 [1]. A similar study to this with a sample of Danish chiropractors to evaluate the use of ICPC-2-DK in chiropractic practice would provide valuable information and potentially ease the implementation process.

## Conclusion

Most of the chiropractors in this study found the use of diagnostic coding both feasible and applicable in their practices but recommended specific training for chiropractors in the use of the coding system. However, there was a strong agreement that the terminology in ICPC-2 PLUS would not be applicable or desirable for those members of the chiropractic profession who do not focus on symptoms in their approach to clinical care. Clinically relevant research opportunities and further integration in the mainstream health care system were mentioned as some of the future perspectives of diagnostic coding.

Thus, this first insight into the use of diagnostic coding in chiropractic practice is encouraging, but more research is needed to determine if these results are applicable to chiropractic practice in other countries and to investigate possibilities for improving the chiropractic relevance of the terminology.

## Additional files

Additional file 1:
**The encounter form.**
Additional file 2:
**Additional quotes.**
