# Supplementary material for: The use of diagnostic coding in chiropractic practice
Source: Chiropr Man Therap. 2015 Feb 18;23:8. doi: 10.1186/s12998-015-0051-1 (PMC4333265; doi:10.1186/s12998-015-0051-1)
Supplement: Additional file 2: — Additional quotes. [file 12998_2015_51_MOESM2_ESM.docx]

# Additional quotes

| **Overall attitudes**  *“Absolutely, yeah. I think it would be brilliant.”* [ID9, 48 years old, 22 years in practice, Metro]  *“It’s like trying to fit a square peg into a round hole”*[ID11, 52 years old, 19 years in practice, Rural]  *“For third-party payers, potentially. In private practice, not relevant. It just doesn't really - it's not needed.”* [ID10, 49 years old, 22 years in practice, Metro]  ***Advantages and disadvantages of using a clinical coding system in chiropractic practice***  *“I see the function it can serve as being as an excellent tool for searching for cases and that function, yeah, I see it obviously goes a fair way towards doing that. It’s something that’s being adopted in other healthcare fields. No, it sounds like an excellent instrument that can bring some level of efficiency to the task of research.”* [ID14, 56 years old, 32 years in practice, Rural]  *“It’s very symptom based and it doesn’t fit well with a wellness based model”* [ID13, 30 years old, 5 years in practice, Metro]  *“There is no doubt it* [ICPC-2 PLUS] *has got applications for musculoskeletal manual therapy the benefits of it, which is one of the issue we have had within the discipline is the terminology and the variation in terminology. So you can ask 10 different practitioners to describe the same thing and we can go from spinal segment motion dysfunction to subluxation to mechanical back pain to facet joint restriction, the list goes on. So I think if there was continuity and streamlining of that terminology it would remove some of the confusion that exists not only within the discipline but also communication externally as well.”*[ID1, 48 years old, 16 years in practice, Metro]  *“I’d say this kind of system would help put us in touch with, let’s say, the medical profession. That would be something that they would recognise more so it means that we’re no longer people with tree heads, you know. Maybe the stuff that they pick up from this kind of coding would allow them to understand at least what we’re looking at.”* [ID2, 50 years old, 28 years in practice, Metro]  ***ICPC-2 PLUS terminology issues for chiropractic practice***  *“It’s just overlapping. The terminology's all right. It's just you could take one condition which would then probably fitted into half a dozen of those in some cases.”* [ID12, 38 years old, 16 years in practice, Rural]  *“The second bit of it, the coding bit, almost too open-ended. There are just too many choices, too much.”* [ID10, 49 years old, 22 years in practice, Metro]  *“There's obviously a lot of overlap there and I guess you've just got to pick one that best encapsulates all of them.”* [ID12, 38 years old, 16 years in practice, Rural]  *“Really that doesn’t tell me exactly what I did, that wouldn’t necessary confer with what I have written in my files”*[ID2, 50 years old, 28 years in practice, Metro]  “*One or two of the descriptors there make me think oh, I’d like to read their definition of what cervical syndrome, thoracic lumbar syndrome would be. So some elaboration on some of the descriptor terms so we’re working on the same page, so to speak. Otherwise I would probably shy away from using those terms that potentially are a big description that’s not fully elaborated*” [ID14, 56 years old, 32 years in practice, Rural]  *Implementation of a coding system into chiropractic practice*  “*Once I got my head around it, it was quite fun, and interesting*.”[ID9, 48 years old, 22 years in practice, Metro]  *“It is a bit challenging, just not being use to it”* [ID4, 57 years old, 26 years in practice, Rural]  “*It wouldn't be challenging. It would probably just take a little bit of getting used to. But I think after a few weeks of getting to know the codes and knowing what you see, you'd get the hang of it pretty quick.*” [ID13, 30 years old, 5 years in practice, Metro] |
| --- |
